# Clinical Ethics Consultations in the Opinion of Polish Physicians

**DOI:** 10.1007/s11673-021-10116-0

**Published:** 2021-08-16

**Authors:** Marek Czarkowski, Joanna Różyńska, Bartosz Maćkiewicz, Jakub Zawiła-Niedźwiecki

**Affiliations:** 1 Collegium Medicum, Cardinal Stefan Wyszyński University, Kazimierza Wóycickiego 1/3, 01-938 Warszawa, Poland; 2 University of Warsaw, Krakowskie Przedmieście 3, 00-927 Warszawa, Poland

**Keywords:** Clinical ethics consultation, Physicians, Decision-making, Poland

## Abstract

Clinical Ethics Consultations (CEC) are an important tool for physicians in solving difficult cases. They are extremely common in North America and to a lesser extent also present in Europe. However, there is little data on this practice in Poland. We present results of a survey of 521 physicians practising in Poland concerning their opinion on CECs and related practices. We analysed the data looking at such issues as CECs’ perceived availability, use of CECs, and perceived usefulness of such support. Physicians in our study generally encounter hard ethics cases, even—surprisingly—those who do not work in hospitals. Most physicians have no CEC access, and those that do still do not employ CECs. However, physicians perceive this form of support as useful—even more so among actual users of CECs. We compared these findings with similar studies from other European countries and the North America. We point out peculiarities of our results as compared to those in other countries, with some possible explanations. We hope the results may encourage regulatory debate on the need to formally introduce CECs into the Polish healthcare system.

## Introduction

Clinical ethics consultations (CECs) are one of the central components of clinical ethics support. CECs assist patients, families, and all medical professionals in defining, analysing, and solving ethical dilemmas accompanying healthcare (Orr and Moon 1993; McClung et al. 1996; Orr et al. 1996; Schneiderman et al. 2006; Arnold et al. 2011). Depending on the model implemented in each case, consultations are provided by individual consultants, small groups, or committees (La Puma and Toulmin 1989; Singer et al. 1990; Rushton et al. 2003). In Europe, CECs are usually offered by Hospital Ethics Committees (HECs) (UNESCO 2006; Larcher et al. 2010; Richter 2014; Schochow et al. 2016; Schochow et al. 2019). Newer forms of ethics support exist as well e.g. Moral Case Deliberation (Stolper et al. 2016) or Ethics Reflection Groups (Lillemoen and Pedersen 2015), as well as others (Hartman et al. 2020). However, there is no evidence of such methods being applied in Poland.

Many divergent factors affect the availability and use of CECs in specific countries and healthcare facilities. One of the universal factors is a growing complexity (technical, institutional, social, and ethical) of modern clinical practice that often leads to medical professionals feeling lost and in need of independent ethical expertise (Aulisio 2003; Moreno 2010). Indeed, research shows that physicians represent a professional group that is the most interested in having access to such services and the most willing to use them (Fox et al. 2007; Swetz et al. 2007). However, it does not mean that there are no differences in physicians’ declarations regarding their need for CECs (Orlowski et al. 2006). At a local level, the availability and use of CECs is determined by various political, institutional, and social factors, in particular: local culture, trust relations, dominating model of patient-physician relationship, and existing legal and administrative frameworks (Gefenas 2001; Borovečki et al. 2005; Vollmann 2013). In many countries, e.g. in the United States (Votano et al. 2004; Swetz et al. 2007) and in Poland (Center for Health Care Quality Monitoring 2016; *Act of 6 November* 2008*on accreditation in healthcare*) CECs have been introduced to healthcare facilities as a hospital accreditation standard. However, the implementation level of those standards varies. In Poland, there is no legal requirement for meeting the accreditation standards and consequently, for establishing CECs in healthcare facilities. Moreover, mere establishment of CEC services in healthcare facilities often does not imply that physicians working in these facilities actually have access and/or are willing to use CECs, as these may exist “on paper” only. This seems to be the case also in Poland, as we have been unable to identify evidence of CEC activity in the majority of healthcare institutions. However, there is no scientific data that would prove the point. A major goal of this paper is to fill the gap by investigating Polish physicians’ knowledge and perception of CECs.

We report the results of the very first survey of Polish physicians’ opinions regarding the availability of CECs and their experience with using CECs in the process of making tough clinical decisions, where “tough clinical decisions” are broadly defined as decisions about the patient’s treatment or care that respondents considered difficult to make for ethical reasons or other reasons not directly related to the respondents’ medical knowledge or skills. The term was deliberately left imprecise to allow flexibility of interpretation. It was assumed, however, that such decisions form an integral part of everyday healthcare practice of all physicians worldwide, including Polish doctors, and they may be related to a variety of factors, such as conflicting values or interests between healthcare professionals and patients or their relatives, problems in interpersonal relations between the parties, organizational issues, or legal requirements (Kälvemark et al. 2004; Hurst et al. 2007; Rasoal et al. 2016). Tough clinical decisions cause medical professionals to experience unease or uncertainty about what is right or good to do and how to act in a given situation (Rasoal et al. 2016).

The results presented herein are one part of a larger study aimed at investigating how Polish physicians deal with such decisions, conducted in 2018–2019 by researchers from the Center for Bioethics & Biolaw of the University of Warsaw and Collegium Medicum of Cardinal Stefan Wyszyński University in cooperation with the Center for Bioethics at the Polish Chamber of Physicians and Dentists. The study addressed the following questions:

1. Do Polish physicians face tough clinical decisions?
2. Do Polish physicians have access or know about access to CECs in their place of work?
3. How often do they use CECs when confronted with tough clinical decisions?
4. Do they find CECs helpful in dealing with tough clinical decisions?

## Methods

### Survey Questionnaire Development and Design

As mentioned above, this paper presents the results of one part of a larger empirical study on how Polish physicians deal with tough clinical decisions, conducted in 2018–2019. For the purpose of the study, we developed a questionnaire composed of 17 close-ended questions using quasi-Likert 5 level scales, from which three included answers labelled “other; please specify.” Some of the questions were interrelated; two were quite long and complex, as they contained additional sub-questions. (The presentation of answers to these more demanding questions must be left for another occasion as these warrant paper-sized analysis each.)

The questionnaire was drafted based on a broad review of the ethics consultation literature. However, the main inspiration for the content of our survey instrument came from the study of U.S. internists’ experiences with ethical dilemmas and ethics consultation, conducted by Gordon DuVal et al. (DuVal et al. 2004). Similar to DuVal’s team’s survey, our questionnaire contained questions related to the following domains: (1) respondents’ professional qualifications, experience, and practice characteristics (localization and type of healthcare facility); (2) physicians’ experiences with facing tough decisions in clinical practice (frequency, medical context); (3) description of main ethical (and other non-medical) reasons that contributed to the perceived toughness of the clinical decisions; (4) methods employed by respondents to deal with the encountered tough clinical decisions and their usefulness; (5) respondents’ education in medical ethics and its importance in addressing tough clinical problems; and the domain essential for this paper that contained questions regarding (6) respondents’ experience with and opinions about CECs, including their accessibility, use, and perceived helpfulness.

The questionnaire was designed to be self-administered, i.e. completed by respondents without the intervention of the researchers collecting the data. To evaluate its feasibility, readability, accuracy, and duration, a small pilot study was conducted. Fifteen physicians took part in it. Results of the pilot study were used to improve the questionnaire design and wording. The number and length of questions were reduced in order to meet the respondents’ expectation that it should take no more than ten minutes to complete the survey.

The questionnaire was developed in the Polish language. Relevant questions and results have been translated into English for the purpose of this report. Any disagreements regarding the translation were resolved by consensus between three researchers responsible for the development of the original questionnaire. All the researchers have extensive educational or working experience in English-speaking environments.

### Survey Distribution and Data Collection

The survey was advertised to all 150,000^1^ Polish physicians licensed to practise. Therefore, information about the study was disseminated widely via “Gazeta Lekarska” (Medical Newspaper)—a journal issued both in online and print format by the Polish Chamber of Physicians and Dentists (as one of its statutory duties) and sent free of charge to all licensed Polish doctors (Gazeta Lekarska 2018).

We used two media of distribution of the survey questionnaire: online and on paper. A link to the online version was included in the information about the study published in “Gazeta Lekarska.” It was also made available through the Newsletter of the Polish Chamber of Physicians and Dentists accessible to all Polish doctors with license to practice. The link was also placed on the official website of the Center for Bioethics & Biolaw. Paper questionnaires were handed to consenting physicians during selected medical and bioethics conferences, as well as during post-graduate training courses. They were also distributed in hospitals.

The survey was conducted between June 2018 and February 2019. In total, 521 completed questionnaires were collected. More than two thirds were filled online (n = 389), and almost one third were submitted on paper.

### Respondents

In total, 521 physicians completed the survey. Almost all respondents had already obtained specialization at least in one medical specialty (43.8 per cent, n = 228; further referred to as specialists) or were undergoing a residency programme to become a medical specialist at the time of the study (50.1 per cent, n = 261; further referred to as residents). Only 32 (6.1 per cent) respondents had an M.D. degree without additional education or training in any medical specialty (further referred to as “non-specializing physicians”).

Most main medical and surgical specialties were represented in the sample (both among specialists and residents). Due to the large number of official specialties in Poland, we grouped them into several major categories as follows (residents in brackets): internal medicine 63 (72), anaesthesia and intensive care 41 (24), paediatrics 98 (34), psychiatry 16 (15), family medicine 12 (22), surgical specialties including gynaecology 36 (61), other (mostly diagnostic, i.e., pathology, radiology, laboratory medicine) 17 (30). Due to multiple specialty holders, these do not sum up to 521. Since our respondents were not selected according to their specializations—in other words, our sample was an opportunity sample—respondents’ specialties will not be reported or analysed further.

The average seniority in the sample was 11.24 years (SD = 10.55, MED = 7). The surveyed physicians had from less than a year to fifty-four years of professional experience. The majority of respondents, 79.6 per cent (n = 413), had less than twenty years of seniority, and only 20.4 per cent (n = 106) physicians had seniority greater than twenty years (two respondents did not provide answer to this question). Thus, there is an overrepresentation of younger practitioners in the sample, most probably due to the mixed method of questionnaire distribution—online and on paper. It is interesting to note that the average professional experience of the respondents in pen and paper mode was significantly longer than of the respondents in the online mode: 10.6 years (SD = 10.4, MED = 7), as compared to 13.1 years (SD = 10.7, MED = 8), Mann-Whitney U: U = 21216, p = 0.0036.

Data on the respondents’ age and gender were not collected. Questions related to them had been removed from the final version of the questionnaire in response to the pilot study results indicating that the collection of those data could make some respondents identifiable e.g. in case of physicians with two or more medical specialties.

The vast majority of responding physicians worked in inpatient healthcare facilities, such as hospitals, hospices, nursing homes, social long-term care homes (86.6 per cent; n = 451). Doctors practising in hospitals corresponded to 82.7 per cent of the sample (n = 431). Almost half of the sample worked in outpatient settings, for example, clinics or private medical offices (45.7 per cent; n = 238). However, only a small number of respondents worked exclusively in outpatient healthcare settings (13.4 per cent; n = 70). These data do not sum up to 100 per cent due to the fact that many physicians are multiple job holders. They practise simultaneously in inpatient and in outpatient settings.

The majority of the surveyed physicians (71 per cent, n = 370) reported that their main place of clinical practice was located in a city with 200,000 or more inhabitants, 14.7 per cent (n = 77) worked in cities with population between 50,000 and 200,000, and 12.1 per cent (n = 63) in cities with 5,000 to 50,000 inhabitants. Only 0.9 per cent (n = 5) worked in towns of less than 5,000 inhabitants and 1.1 per cent (n = 6) of the sample worked in villages, which can be explained to some extent by the used sampling method.

### Data Analysis

The online survey was run using the Limesurvey software. All data analysis was carried out using the R programming environment. The data were analysed using descriptive statistics, Fisher exact test, Mann-Whitney U test, and Spearman rank correlation, as appropriate. Two-tailed tests were used. The test used and the significance are indicated together with the corresponding results. “SD” is used for standard deviation, “M” for mean and “CI” for 95% per cent confidence interval, where appropriate.

### Ethical Considerations

The survey did not involve the collection of personally identifiable information (personal data). Participation was anonymous and fully voluntary. The Research Ethics Committee at the Faculty of Sociology of the University of Warsaw approved the study.

## Results

The survey reveals that the majority of Polish physicians 76.6 per cent (n = 399) out of all those who took part in the study (n = 521), faced tough clinical decisions in their professional practice. However, controlling for experience, the odds of encountering tough clinical decisions are 2.34 higher in the specialists group compared to residents and “non-specializing physicians.” Physicians who worked only in outpatient settings (13.4 per cent of the sample; n = 70) dealt with difficult healthcare decisions less frequently than their inpatient peers (65.7 per cent vs 78.3 per cent, Fisher exact test: p = 0.022).

### Perceived CEC Availability

Since CECs are typically provided in inpatient healthcare facilities, for the analysis of the perceived availability and use of CECs, we excluded respondents who worked exclusively in outpatient settings.

Out of the 451 physicians practising in inpatient settings, only 287 (63.6 per cent) were able to give a positive or negative answer to the question whether CECs were provided in their healthcare facilities. Almost half of them (49.2 per cent; n = 222) reported that CECs were not available in their healthcare facilities; whereas only 14.4 per cent (n = 65) confirmed awareness of CECs being provided in their facilities. More than one third of the inpatient physicians (33.9 per cent, n = 153) admitted that they had no knowledge on whether CECs were provided in their places of practice, and eleven inpatient physicians (2.4 per cent) did not provide any answer to this question.

Knowledge about the availability of CECs differed among respondents depending on the level of their professional education and training, as well as their professional experience (hence, presumably also age). Close to two thirds of all inpatient “non-specializing physicians” (60 per cent; n = 18 [out of 30]) did not know whether CECs were available in their healthcare facilities, while in the groups of inpatient residents and specialists the numbers of not-knowing respondents were significantly lower: 41.8 per cent (n = 99 [out of 237]) and 19.6 per cent (n = 36 [out of 184]), respectively (Fisher exact test: p < 0.001). Professional experience (determined by the years of practice) also significantly predicted knowledge about the availability of CECs (logistic regression: β = 0.062, z = 5.033, χ(1) = 25.33, p < 0.001).

### Use of CECs

As noted earlier, only few surveyed physicians working in inpatient settings declared knowledge of having access to these services (14.4 per cent; n = 65). Among those, less than half had actually ever used CECs. Due to the sparsity of data, inpatient physicians’ responses regarding how often they sought CECs assistance when confronted with tough clinical decisions were grouped into three options. The survey revealed that 60 per cent (n = 39) of doctors who knew of CEC availability had never asked for this kind of support (this group also includes physicians who did not answer this question but declared awareness of having access to CECs); 30.8 per cent (n = 20) used CECs at least once in their career, and only 9.2 per cent (n = 6) always request CECs assistance (both groups are exclusive of each other). Thus, in total, 40 per cent (n = 26) of the surveyed inpatient physicians who had access to CECs in their healthcare facilities had any experience using the services (further referred as CEC-users). This means that among all inpatient physicians only 5.7 per cent had used CEC services.

### Perceived Helpfulness of CECs

Over a half of all the surveyed physicians (55.1 per cent; n = 287) agreed or strongly agreed with the general statement that the availability of CECs is helpful or would be helpful (if provided) in their everyday healthcare practice, while 23.4 per cent of the respondents (n = 122) disagreed or strongly disagreed with the statement. The remaining 21.5 per cent of the sample (n = 112) explicitly stated that they had no opinion on CEC’s helpfulness or did not provide any response. For the purpose of further statistical analysis, we coded respondents’ answers to the statement as numbers ranging from 1 (strongly disagree) to 5 (strongly agree). We found that physicians who faced tough clinical decisions in their professional practice consider CECs to be more helpful (M = 3.54, SD = 1.10, CI 95 per cent [3.43–3.65]) than those who never faced such decisions (M = 3.24, SD = 1.2, CI 95 per cent [3.02–3.47], Mann-Whitney U test: U = 18982, p = 0.022). We did not find statistically significant correlation between the frequency of facing tough clinical decision and the opinion on the helpfulness of CECs (Spearman ρ = 0.077, p = 0.08).

In the group of respondents working in inpatient settings (n = 451), the general opinion on CECs was slightly more positive than in the total sample. 57.4 per cent (n = 259) of inpatient physicians agreed or strongly agreed with the statement that the availability of CECs is helpful or would be helpful (if provided) in their everyday healthcare practice, 21.9 per cent (n = 99) disagreed or strongly disagreed, and 20.6 per cent (n = 93) explicitly stated that they had no opinion on CEC’s helpfulness, or did not provide any response. We also compared opinions on the helpfulness of CECs between inpatient specialists, residents and “non-specializing physicians” (coded as numbers ranging from 1 [strongly disagree] to 5 [strongly agree]). We found statistically significant differences between these three groups (Kruskal-Wallis: χ(2) = 8.14, p = 0.017). The mean response in the specialist group was 3.68 (SD = 1.10, 95 per cent CI: 3.51–3.84), whereas in the group of “non-specializing physicians” the mean was 3.07 (SD = 1.20, 95 per cent CI: 2.62–3.52, Mann-Whitney U [with Holm adjustment]: U = 1879, p = 0.024). We did not find statistically significant differences between residents’ and specialists’ opinions.

Even more positive were the general opinions on the helpfulness of CECs of those respondents who practiced in settings where CECs were available. For the purpose of this analysis, we defined a group of “potential CEC-users”: to the sixty-five inpatient physicians who confirmed that CECs are available in their healthcare facilities we added two outpatient respondents who declared that they had access to CECs. (We assumed that they did not currently work in an inpatient service but either had worked there before or had access to CECs through other means such as an academic appointment.) We compared answers provided by the “potential CEC-users” (n = 67) with those provided by all other physicians in the sample. For the purpose of statistical analysis, we also coded respondents’ answers to the statement as numbers ranging from 1 (strongly disagree) to 5 (strongly agree). Mean agreement with the statement about helpfulness among “potential CECs-users” was 3.79 (SD = 1.08, CI 95 per cent [3.53–4.05]) whereas the rest of the sample rated CECs’ helpfulness significantly lower (no CEC available: M = 3.40, SD = 1.18; do not know whether CEC is available: M = 3.48, SD = 1.05). There were statistically insignificant differences in opinions of those respondents who had knowledge about the unavailability of CECs (M = 3.40, SD = 1.18, CI 95 per cent [3.26–3.54]) and those who had no knowledge on CECs availability (M = 3.48, SD = 1.05, CI 95 per cent [3.32–3.64]).

The group of “CEC-users” (n = 26; 5 residents, 21 specialists) tended to agree even more strongly with the claim that CECs are helpful or can be helpful (M = 4.04, SD = 0.96, CI 95 per cent [3.65–4.43]) than non-users taken as a whole (M = 3.51, SD = 1.13, CI 95 per cent [3.40–3.61], Mann-Whitney U test: U = 3975, p = 0.02).

## Discussion

The size of our sample was higher or close to other studies on similar topics (DuVal et al. 2001; DuVal et al. 2004; Orlowski et al. 2006). As opposed to that other research, in response to results of the pilot study, we did not collect data on age and gender.

Over three quarters of Polish physicians have faced tough clinical decisions in their professional practice, i.e. situations when they had a problem with making a clinical decision for reasons other than strictly medical. The population surveyed by us was more varied than in comparable studies from other countries, as it was not restricted to specific specializations. It can still be observed that the incidence of reported problems was lower than in other countries. As much as 85 per cent of Swedish general practitioners faced ethical issues every week or even more often (Bremberg and Nilstun 2001). In the study of European general practitioners, family physicians, and internists, the incidence of ethical problems was even higher (99.4 per cent), but this result was not obtained from a direct question but rather as a sum of various difficulties chosen from a list given by researchers (Hurst et al. 2007). Our question concerned the perception of physicians and if they actually notice facing tough clinical decisions in everyday practice. It is possible that physicians encounter ethical problems more often than they realize, and such self-awareness is important for employing conscious ethical decision-making techniques. Also it may be the case that certain ethical dilemmas experienced by physicians in other countries are not encountered or reported by their Polish peers simply because Polish law bans or does not regulate certain procedures legally recognized in many Western European countries. For example, advance directives, including do-not-resuscitate orders, are legally used in Poland only to a very limited extent, while the appointment of a healthcare proxy is not possible at all. Additionally, in the Polish legal system there are clear rules on surrogate decision-making for adults who are not legally incapacitated and consequently do not have an appointed guardian, but—due to health condition or other reasons—are unable to give consent. The law does not recognize family members as surrogates. Only a court is authorized to make medical decisions on behalf of such patients. Thus, the attending physicians—legally speaking—do not have to identify the “right decision-maker” or to discuss the patient’s treatment with family members who might have diverging opinions on this matter. Another factor contributing to the relatively low prevalence of dilemmas reported by Polish physicians may derive from a greater attachment to paternalistic forms of doctor-patient relations. All of these reasons narrow the scope of possible ethical disputes physicians feel safe to consult on with CECs and thus promote only one course of action.

In opposition to commonly shared intuition, our study shows that not only inpatient professionals face tough clinical decisions. Physicians employed exclusively in outpatient services also encounter ethical difficulties in making clinical decisions, although they report them less frequently than inpatient care doctors (65.7 per cent vs. 78.6 per cent).

The study also supports the claim that a vast majority of the 76.6 per cent of surveyed physicians who have faced tough clinical decisions have no access to CECs. This applies to both groups—inpatient and outpatient physicians—although for partially different reasons.

In Poland, only hospitals are officially recommended to provide CECs. Thus, in practice, it is unlikely for those doctors who are not employed in hospitals or in hospitals with outpatient clinics to have access to CECs. Taking into account that there are less than one thousand hospitals and over twenty thousand independent clinics in Poland (Central Statistical Office 2018), it is evident that most outpatient physicians—including those who encounter ethical problems in their practice—cannot expect any type of ethical support. This observation is consistent with the results of a study conducted in four Western European countries, whose authors pointed out the need to ensure access to CECs also in these facilities (DuVal et al. 2001; cf: DuVal et al. 2004; Orlowski et al. 2006; Hurst et al. 2007). It is worth noticing that the problem of the availability of CECs also applies to other outpatient healthcare professionals, e.g. nurses (Karlsson et al. 2013).

Also, physicians working in inpatient environments have very limited access to CECs. Almost half of the surveyed inpatient physicians stated that CECs were not provided in their healthcare facilities, while only 14.7 per cent responded that CECs were available in their workplace.

Despite the fact that the Accreditation Standards for Hospitals prescribe that each hospital should: (i) implement a mechanism for solving ethical problems which should consist of a “group of individuals who enjoy universal confidence—an ethics committee—whom both employees and patients may approach when faced with ethical problems”; and (ii) should inform both employees and patients about a possibility to obtain such HEC assistance (Center for Health Care Quality Monitoring 2016), these standards are not commonly implemented. The exact number of HECs in Poland is unknown, as there is no requirement for HEC registration. Definitely, it is much lower than the number of hospitals, although estimates indicate that the number of HECs is increasing. In 2013, the number of operating HECs was at least 111, while in 2019—at least 229 (Czarkowski et al. 2015; The Center of Bioethics of the Supreme Medical Council 2020). Meanwhile, the number of hospitals did not increase so significantly. In 2012, there were 913 hospitals in Poland, while in 2018 this number grew to 949 (Central Statistical Office 2013; Central Statistical Office 2018). The above data suggest that there should be a HEC in every fourth Polish hospital.

Our survey revealed an interesting discrepancy between the estimated percentage of Polish hospitals with HECs (25 per cent) and the percentage of doctors who admitted that CECs are available in their healthcare facilities (14.7 per cent). The gap can be explained in two (non-mutually exclusive) ways: either some HECs exist only on paper (e.g. in accreditation documents) or not all physicians are aware of HECs’ existence in their place of practice. There is anecdotal evidence supporting the first hypothesis. Our study confirmed only the second one: 33.9 per cent of our respondents did not know whether HEC was operating in their facility (further referred to as “CEC-ignorant physicians”).

The number of “CEC-ignorant physicians” is disturbing, as it may show how little they are integrated into their inpatient work environment (often one of many). On the other hand, such a high percentage of “CEC-ignorant physicians” indicates that the hospitals are not properly fulfilling their obligation, imposed by the Accreditation Standards, to inform employees and patients about the existence of HECs (Center for Health Care Quality Monitoring 2016). It can be expected that since so many physicians are not aware of CECs availability, patients and their families are even less informed about the possibility of receiving HEC support.

It should be stressed that lack of knowledge about the CECs availability is considered to be one of the main reasons why physicians do not use this kind of services (Førde et al. 2008; Pedersen et al. 2009; Sorta-Bilajac et al. 2011). In Norway, HECs actively inform and even organize seminars for hospital personnel, in order to ensure that they know and understand HEC’s role, tasks, and potential, as well as mode of functioning (Pedersen et al. 2009). Our results also suggest that the more knowledge of CECs or the more experience with CECs physicians have, the more likely they are to use this form of ethical support. Half of the physicians from our sample who declared having access to CECs and reported facing tough decisions do use CECs. However, the nominal number of these physicians of 26 does not allow for generalizable conclusions. More studies on this topic are needed. In the United States, where access to CECs has been provided for many years in the vast majority of hospitals, 55 per cent of internists, oncologists, and critical care physicians/pulmonologists request CECs (Fox et al. 2007). European clinicians are less likely to seek ethical support than American doctors, although in the referred study only 38.4 per cent of respondents were at least partly hospital-based (Hurst et al. 2007). Doctors from Central Europe are the least likely to use CECs (Sorta-Bilajac et al. 2011).

Our study also showed that lack of knowledge about the CECs availability positively correlates with a lower level of professional development and shorter seniority of respondents. The highest percentage of “CEC-ignorant physicians” was among “non specializing physicians”, i.e. those who are the youngest, least experienced, and least autonomous in their professional capacity. Physicians with higher professional qualifications and longer seniority usually manage and supervise the work of younger colleagues. They are those to whom younger physicians report and turn for advice, also when faced with though clinical decisions. Thus, the need to find the right solution often rest on the shoulders of more experienced physicians—specialists. And they may actively seek help in this respect, which in turn may lead them to learning about the existence of HEC in a given facility.

Apart from the lack of knowledge and hierarchical decision-making, what are the other reasons why less than a half of the surveyed inpatient physicians who had access to CECs in their healthcare facilities do actually use them?

First of all, Polish Accreditation Standards do not specify the tasks of HECs (Center for Health Care Quality Monitoring 2016). They are defined by the hospital management that sets up such committees. For this reason, there are significant differences in the goals and scope of interests of individual HECs. We have conducted a quick review of a few publicly available internal hospital documents establishing HECs and HECs’ by-laws. And we have found that numerous HECs are established to protect patient’s rights and provide ethical consultations. But there are also many whose role is to ensure good relations between hospital employees, to deal with complaints from patients regarding the quality of service or behaviour of the personnel, or to guarantee a proper provision of services to patients-clients. While the first tasks are typical HEC activities, the others fall within the scope of business ethics (Eiser et al. 1999). This problem has been mentioned before in other studies on the activities of Polish HECs (Czarkowski et al. 2015).

Second, anecdotal evidence, as well as professional and teaching experience of the authors suggest that many Polish physicians remain convinced that clinical problems should be discussed with and solved by medical professionals only; and no advice from other experts, especially non-clinicians, is needed. Probably, some physicians also think that the use of CECs is too time-consuming; others question HEC’s mandate, competence, or the efficacy of the CECs as such, or even believe that the use of CECs will make the clinical situation at hand even more difficult to deal with (DuVal et al. 2004). Nevertheless, numerous studies conducted among non-Polish physicians show that most physicians who actually used CECs were satisfied with the resolution of the clinical situations (Schneiderman et al. 2006; Hurst et al. 2007). Our study also indicates that “CEC-users”, i.e. physicians with personal experience of CECs, have the best opinion on their usefulness, while physicians who work in outpatient settings, and thus have no practical experience with CECs, have the worst opinion. Similar results are seen in the evaluation of CECs in clinical practice by physicians who faced tough clinical decisions in their clinical practice, compared to those who did not encounter such problematic situations. We can hypothesize that as the number of physicians who face tough decisions and who have direct experience with CECs increases, the attitude towards CECs among Polish physicians will also improve. This supposition is corroborated by earlier observations made by researchers from Western Europe and Canada on the role of CECs for the development and importance of ethical support in medical practice (Gaudine et al. 2010; Slowther et al. 2012).

We hope that the results of our study will encourage regulatory debate on the need to formally introduce CECs into the healthcare system in Poland, as well as in other countries that are still lacking these services. Admittedly, further analyses are needed to establish an adequate form and methods of CECs for Polish physicians.

## Limitations

Our study has several limitations. Firstly, the choice of an online survey made it impossible to select a representative sample of Polish physicians (as regards to gender, age, length of practice, specialty, or location of practice). Our population is younger than the population of Polish physicians as a whole. Taking into account that the average age of obtaining medical degree in Poland is twenty-five years, and that the average length of our respondents’ practice was (SD) 11.23 (10.55) years with a median of just seven years, we can assume that the vast majority of the surveyed physicians were below the age of forty-seven. Meanwhile, in 2018, only 54 per cent of all licensed physicians in Poland were younger than fifty-five (Central Statistical Office 2018). However, respondents over the age of forty-seven (i.e. those who have practiced for 22 years or more) were only 17.8 per cent of our sample. It is also worth noting that among the 35,000 Polish physicians over the age of sixty-four (23.5 per cent) there is a substantial, but hard to establish, group of those who do not retire. This is specific for Poland, due to a shortage of physicians, especially specialists, as well as relatively low pensions. It is hard to tell what the exact impact of the overrepresentation of younger respondents on the research results was. Older physicians surely did not have ethics training either during undergraduate or postgraduate or specialization training, as the first regulations enforcing ethics training were only promulgated in 2004 (*Ordinance of Minister of Education and Sport from 18th August 2004 changing ordinances regarding standards of teaching for particular programmes and levels of teaching in higher education, Journal of Laws 2004 No. 194 Pos. 1985.* 2004). Before that, the scope and programme of ethics training was regulated by academic bodies of each medical school (the senate) and it could even be omitted altogether (*Act of 12th September 1990 on higher education Journal of Laws 1990 No. 65 Pos. 385* 1990).

Secondly, our study did not directly ask for age and gender due to anonymity concerns raised by participants of pilot study. Thus, any age-related information is derived indirectly.

Thirdly, in other studies of this kind, only selected specialties were included, and targeted surveys were carried out over the phone or by mail. Our method ruled out the possibility of specialty selection, so the respondents represent a more varied population in terms of specialties. We do not know of any publications that would suggest a negative influence of a larger variety of specializations on the quantity of ethical issues experienced by physicians. Moreover, our method of sample selection provides a larger variety of experiences, not only regarding most common ethical issues (Konda et al. 2017; Özer et al. 2018). In our sample, even specialties that are usually regarded as “not clinical” reported having ethical conundrums in the course of their practice.

Lastly, a vast majority of our respondents worked in cities. Taking into account that about 15 per cent of medical consultations happen in a rural environment (Central Statistical Office 2018), and physicians working in rural areas amounted to only 1.2 per cent (n = 6) of our sample, we cannot rule out that the underrepresentation of rural physicians has skewed the proportions of outpatient service physicians who encountered ethical issues in their practice.

## Conclusions

1. The majority of Polish physicians face tough clinical decisions—i.e. problems with making clinical decisions due to ethical or other non-medical reasons—in their daily practice.
2. Many physicians, including those working in inpatients settings, have no access to CECs.
3. Many physicians have no adequate knowledge about the CECs availability.
4. Few of the physicians who face tough clinical decisions ask for CECs.
5. Polish physicians consider CECs as helpful or possibly helpful if they had access.
6. Physicians who used CECs are good advocates for this form of ethical support.

## Footnotes

1 The number of physicians in Poland is a highly contested value with vast differences between sources. The number quoted herein comes from the official register of physician licenses (Centralny Rejestr Lekarzy - Informacje Statystyczne 2021), which includes those who are not practising or are living abroad, because all licenced physicians are supposed to receive the journal we used, as well as the newsletter of the Polish Chamber of Physicians and Dentists.
